# Supplementary material for: The length of a scroll: Quantitative evaluation of material reconstructions
Source: PLoS One. 2020 Oct 21;15(10):e0239831. doi: 10.1371/journal.pone.0239831 (PMC7577460; doi:10.1371/journal.pone.0239831)
Supplement: S1 File — (PDF) [file pone.0239831.s001.pdf]

S1 Appendix: Spiral approximation

The previous discussion used an approximation of concentric circles for computing the length of a scroll. A more precise approximation is that of a spiral. The spiral's advantage is that it is more accurate; its disadvantage is that computing its length is considerably more complicated than computing the length using the sum of concentric circles (as done in Section]Circle Approximation). Compare Figs 3A,B.

As the improvement in precision turns out to be minuscule compared with the uncertainties of the measurements involved, it is not worthwhile taking the more complicated route outlined here. It is true that twenty concentric circles, for example, each 2 mm apart, have a total length of almost 264 cm, while twenty turns of a spiral are only 251 cm long, amounting to a non-negligible difference of over 12 cm, or 5%. (With fewer turns, the percentage is higher, but in absolute terms the difference is less.) All the same, in the context of estimating the remainder of a scroll, this difference does not pose an actual problem. In our example, the last turn would have circumference $c = 24.5$, not 25.1, as one would expect were the scroll really made of 20 concentric circles. The calculated sum of nested circles ($z = 1.3$) ends up being 251, almost exactly like the spiral. It bears stressing that although a spiral is closer to the actual shape of a scroll, it too is only a rough approximation, since historical scrolls are not evenly rolled. Nevertheless, we give here the details of the spiral method, offering scholars the choice according to their specific circumstances and needs.

Unlike for circles, the lengths of consecutive turns do not increase perfectly linearly, even if we accept Assumption C that the radii do. Rather, the difference z in the size of turns grows as the turn gets further away from the center.

The polar equation of a spiral is

$$r(\theta) = r_0 + \rho\theta$$

where r_0 is the distance from the center of the spiral to its starting point and ρ is the rate of increase in radius per radian. (A circle has 2π radians, each radian roughly 57.2°.) The increase in radius in a full turn is $r = 2\pi\rho$. The length L of such a spiral is

$$L = \int_0^{2\pi n} \sqrt{(r_0 + \rho\theta)^2 + \rho^2}\, d\theta$$

where n is the number of turns and can be fractional.

To simplify the development, let's consider first the $r_0 = 0$ case. Now

$$K(\kappa) = \int_0^{\kappa} \sqrt{(\rho\theta)^2 + \rho^2}\, d\theta = \rho \int_0^{\kappa} \sqrt{\theta^2 + 1}\, d\theta$$

gives the length of a spiral starting from the center and completing an angle of κ radians. The solution to the indefinite integral is

$$\int \sqrt{\theta^2 + 1}\, d\theta = \frac{1}{2}\left[\theta\sqrt{\theta^2 + 1} + \sinh^{-1}\theta\right]$$

where $\sinh^{-1} x = \ln(x + \sqrt{x^2 + 1})$ is the inverse (area) hyperbolic sine function and ln is the natural (base e) logarithm. Hence

$$K(\kappa) = \frac{\rho}{2}\left[\kappa\sqrt{\kappa^2 + 1} + \sinh^{-1}\kappa\right]$$

Letting $\kappa = 2\pi n$, this is

$$L(n) = K(2\pi n) = \rho\left[\pi n\sqrt{4\pi^2 n^2+1} + \tfrac{1}{2}\sinh^{-1}(2\pi n)\right] \tag{12}$$

If the scroll is κ radians in toto, then the length of the outermost turn is

$$c = K(\kappa) - K(\kappa - 2\pi)$$

So, z, the increment in length of the outermost turn over the prior one, is the difference between the outermost and the next one in:

$$\begin{aligned}
z &= [K(\kappa) - K(\kappa-2\pi)] - [K(\kappa-2\pi) - K(\kappa-4\pi)] \\
&= K(\kappa) - 2K(\kappa-2\pi) + K(\kappa-4\pi) \\
&= \frac{\rho}{2}\Big[\quad (r/\rho)\sqrt{(r/\rho)^2+1} + \sinh^{-1}(r/\rho) \\
&\qquad - 2(r/\rho-2\pi)\sqrt{(r/\rho-2\pi)^2+1} + \sinh^{-1}(r/\rho-2\pi) \\
&\qquad + (r/\rho-4\pi)\sqrt{(r/\rho-4\pi)^2+1} + \sinh^{-1}(r/\rho-4\pi)\Big]
\end{aligned}$$

where $r = \rho\kappa$ is the radius of the outermost edge of the scroll. The value of z increases slowly as r grows, especially when loosely rolled (and ρ is large).

The problem is that we know neither ρ nor n. We may suppose that we know the length c of the outermost turn and the difference z of the lengths of the outermost two turns. Still, given z, there is no (obvious) way to solve for ρ analytically. All the same, since the approximation of a spiral differs only slightly from the approximation of concentric circles when it comes to one or two turns, we can estimate n and ρ based on the circle model:

$$n \approx \frac{c}{z} \qquad\qquad \rho \approx \frac{z}{4\pi^2}$$

Then equation (12) yields

$$\begin{aligned}
L &\approx \frac{z}{4\pi^2}\left[\frac{\pi c}{z}\sqrt{4\pi^2\left(\frac{c}{z}\right)^2+1} + \frac{1}{2}\sinh^{-1}\left(2\pi\frac{c}{z}\right)\right] \\
&= \frac{c}{2}\sqrt{\frac{c^2}{z^2}+\frac{1}{4\pi^2}} + \frac{z}{8\pi^2}\sinh^{-1}\left(\frac{2\pi c}{z}\right)
\end{aligned}$$

The above approximation fits most turns of the spiral, but in the innermost parts the spiral starts much quicker than concentric circles. Thus a better approximation for n is

$$n \approx \frac{c}{z} + \frac{1}{2}$$

Plugging this n into (12), we get

$$L \approx \frac{z}{4\pi^2}\left[\pi\left(\frac{c}{z}+\frac{1}{2}\right)\sqrt{4\pi^2\left(\frac{c}{z}+\frac{1}{2}\right)^2+1} + \frac{1}{2}\sinh^{-1}\left(\frac{2\pi c}{z}+\frac{1}{2}\right)\right] \tag{13}$$

which is how the penultimate column in Table 2 was computed. Without smaller terms this is

$$L \approx \frac{c^2}{2z} + \frac{c}{2} + \frac{z}{6.4} + \frac{z}{79}(\ln c - \ln z) \tag{14}$$

which is close to (11).

Better yet, one can search for a close numerical approximation by repeatedly estimating n and ρ and checking

$$\begin{aligned} c' &= L(n) - L(n-1) \\ z' &= L(n) - 2L(n-1) + L(n-2) \end{aligned}$$

using the estimate for ρ. If c' is sufficiently close to the measured value of c and z' to z, then we're done. If not, then the estimates of n and ρ can be improved by

$$\begin{aligned} n &:= n + (c - c')/z \\ \rho &:= \rho + (z - z')/(4\pi^2) \end{aligned} \tag{15}$$

after which one repeats the steps. This gives the very precise values in the last column of Table 2. This more accurate spiral length, however, has negligible overall impact on the results of the calculations. In particular, Table 1 is unaffected.

If one does need to take a core of radius r_0 into account, then, letting $\kappa_0 = r_0/\rho$, the desired length estimate would be

$$\begin{aligned} L &= K(\kappa_0 + 2\pi n) - K(\kappa_0) \\ &= \frac{\rho}{2}\left[(2\pi n + \kappa_0)\sqrt{(\kappa_0 + 2\pi n)^2 + 1} - \kappa_0\sqrt{\kappa_0^2 + 1} + \sinh^{-1}(\kappa_0 + 2\pi n) - \sinh^{-1}\kappa_0\right] \end{aligned}$$

Finally, given ρ and L, one may want to invert the spiral formula and solve for n $(= \kappa/(2\pi))$, again ignoring any core (letting $\kappa_0 = 0$). We have

$$\begin{aligned} L &= \frac{\rho}{2}\left[\kappa\sqrt{\kappa^2+1} + \ln\left(\sqrt{\kappa^2+1} + \kappa\right)\right] \\ &\approx \frac{\rho}{2}\left[\kappa\sqrt{\kappa^2} + \ln\left(\sqrt{\kappa^2} + \kappa\right)\right] = \frac{\rho}{2}\left[\kappa^2 + \ln\kappa + \ln 2\right] \end{aligned}$$

Solving the last expression for L gives

$$\kappa = \sqrt{\tfrac{1}{2}W\left(\tfrac{1}{2}e^{4L/\rho}\right)}$$

where W is the Lambert (omega, product log) function, and, therefore,

$$n = \frac{1}{2\sqrt{2}\pi}\sqrt{W\left(\tfrac{1}{2}e^{4L/\rho}\right)} \tag{16}$$

Approximating further, taking $W(x) \approx \ln x$ (albeit it's closer to $\ln x - \ln\ln x$), we arrive at

$$n \approx \sqrt{\frac{L}{\pi s}}$$

recalling that $s = 2\pi\rho$. Compare this with equation (9) for the circle approximation. For example, with $L = 5027$ and $s = 1$, both formula (16) and this simple approximation yield the correct value 40.00 for n.

S2 Appendix: Wedge approximation

A somewhat different method of approximation has been used for similar purposes by papyrologists. Often only a wad of sheets remains from a biblion (papyrus roll), each layer comprising only a fraction of a

revolution. If, in addition, another matching fragment of a larger, outer layer is found, then one wants to estimate the length of the missing part between the wad and the additional fragment. See S1 Fig . In this section, we follow the method expounded by Essler [34, §4.2]. (Cf. [35, p. 149].)

S1 Fig. Wedge-based approximation.
Wedge arc of 115° (about 2 radians). (A) Left: layers with radii $r = 0.5, 0.6, \ldots, 2.5$. The red solid parts are preserved; the black broken area is to be reconstructed. (B) Center: fraction C of the area. (C) Right: fraction c of the circumference.

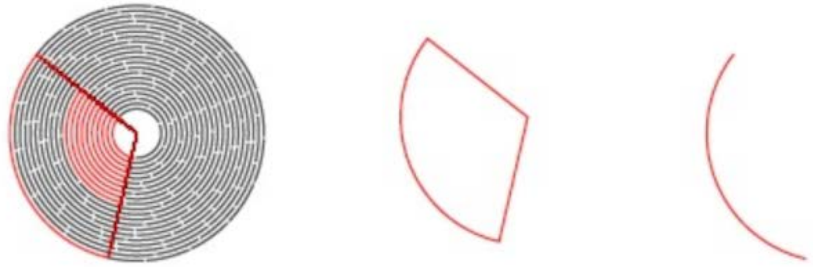

Think of a pie with radius r ($r > 0$). The area C of a wedge spanning a fraction γ of the full circle ($0 < \gamma \leq 1$) is

$$C = \gamma\pi r^2$$

(In a full circle, when $\gamma = 1$, this is πr^2.) The length of the curved arc c along the outer rim of this wedge (the part of the circumference of the whole circle that belongs to the wedge C) is

$$c = 2\gamma\pi r \tag{17}$$

See Figs S1B,C. It follows that

$$C = \frac{c^2}{4\gamma\pi} = \frac{cr}{2} \tag{18}$$

If s is the (nonzero) thickness of a layer (that is, the difference between successive radii), then the (approximate) length ℓ of all the slivers in the wedge laid out is

$$\ell = \frac{C}{s} = \frac{cr}{2s} \tag{19}$$

Given c, ℓ, and s, one can derive $r = 2s\ell/c$, and

$$\gamma = \frac{c}{2\pi r} = \frac{c^2}{4\pi s\ell} \tag{20}$$

Letting L signify the original length of the papyrus roll, not just of the preserved wedge-shaped wad, we have

$$L = \frac{\ell}{\gamma} = \frac{4\pi s\ell^2}{c^2} \tag{21}$$

Now, let a, b, and c be (the lengths of) any three matching segments (not necessarily full turns), all spanning the same angle γ, with $b \neq a$; A, B, C the areas of the respective wedges; ℓ_a, ℓ_b, ℓ_c, the lengths of all the fragments in the corresponding wedges; and L_a, L_b, L_c, the lengths of complete rolls from the center up to (and including) those points. Based on Eqs. (18–21), we infer the following proportions:

$$L_a : L_b : L_c :: \ell_a : \ell_b : \ell_c :: A : B : C :: a^2 : b^2 : c^2$$

and, in particular,

$$\frac{a^2}{L_a} = \frac{b^2}{L_b} = \frac{c^2}{L_c} = 4\pi s\gamma^2$$

By the same token (by 17–21),

$$\frac{c^2 - a^2}{L_{ca}} = \frac{c^2 - b^2}{L_{cb}} = \frac{b^2 - a^2}{L_{ba}} = 4\pi s\gamma^2 \tag{22}$$

where L_{xy} stands for $L_x - L_y$, the approximated length from y to x (including segment x but excluding y). Thus, L_{cb} is the unknown length of the roll from the core until segment c minus the part from the core until and including segment b. This length can be approximated using the measured values. The lengths of the three segments, a, b, and c, are known, as well as the total length ℓ_{ba} of the extant wedge and the thickness s of each of its layers. So we can compute as follows:

$$L_{cb} = L_{ba}\frac{c^2 - b^2}{b^2 - a^2}$$

where $L_{ba} = \ell_{ba}/\gamma = 4\pi s\ell_{ba}^2/a^2$ (per Eq 20).

Turning now to the question of estimating the length of parchment scrolls, the same equations can be used. Suppose b and c are adjacent *full* circumferences ($b \neq c$), so $L_{cb} = c$, and suppose $a = 0$ is the center of the scroll (see Fig 3. We have, using (22) again,

$$L_c = L_{ca} = L_{cb}\frac{c^2 - a^2}{c^2 - b^2} = \frac{c^3}{c^2 - b^2} \tag{23}$$

Note that in this case, with two fully preserved turns, the thickness s plays no role.

The “Wedge” column of Table 2 shows that (23) is reasonably close to our other approximations, but not quite as good, theoretically. This wedge-based approximation L_c is close to the length L'_c, which one gets from the first two terms of (Eq 11) above for circles and of (Eq 14) below for spirals:

$$L'_c \approx \frac{c}{2}\left(\frac{c}{c-b} + 1\right) = \frac{c^3}{c^2 - b^2} - \frac{c}{2}\left(\frac{c^2 - bc}{c^2 - b^2} - 1\right) = L_c + \frac{bc}{2(b+c)}$$

Thus, the wedge method results in a consistently shorter length than the circle method of the main text.

Suppose, now, that b and c are two matching outer *partial* turns (and that $a = 0$), and that nothing else remains of the scroll. Given a presumed value for s, we can in fact use the above formulæ to estimate the total length L_c. Looking back at (23), we obtain that

$$\begin{aligned}
n &\approx \frac{c}{c-b} \\
r &= ns = \frac{cs}{c-b} \\
\gamma &= \frac{c}{2\pi r} = \frac{c-b}{2\pi s} \\
L_{cb} &= \frac{c}{\gamma} = \frac{2\pi cs}{c-b} \\
L_c &= L_{cb}\frac{c^2}{c^2 - b^2} = \frac{2\pi sc^3}{(c-b)^2(b+c)}
\end{aligned} \tag{24}$$

When b and c are complete turns, $c - b = 2\pi s$, and this formula for L_c reverts back to (23).

Using the concentric-circle estimation method of the main text (Eq 7), rather than this wedge method, and denoting by $b_\circ$ and $c_\circ$ the unknown lengths of the two original full turns, we arrive at a somewhat better estimate than (24) for this case of partially preserved turns:

$$\begin{aligned}
c_\circ &= \frac{c}{\gamma} = \frac{2\pi sc}{c-b} \\
b_\circ &= \frac{b}{\gamma} = \frac{2\pi sb}{c-b} \\
L_c &= \frac{c_\circ}{2}\left(\frac{c_\circ}{c_\circ - b_\circ} + 1\right) = \frac{\pi sc}{c-b}\left(\frac{c}{c-b} + 1\right)
\end{aligned}$$
