# Supplementary material for: The length of a scroll: Quantitative evaluation of material reconstructions
Source: PLoS One. 2020 Oct 21;15(10):e0239831. doi: 10.1371/journal.pone.0239831 (PMC7577460; doi:10.1371/journal.pone.0239831)
Supplement: Attachment — Submitted filename: Response to Editors.docx [file pone.0239831.s002.docx]

Response to Editors

The Length of a Scroll

We thank the editor for his comments, and propose the following clarifications:

1. "When you list three assumptions on page 4, I don’t see the most important assumption that there are no missing pieces at the end of the scroll. What if the scroll you analyze is just the first part of a longer scroll? Moreover, missing parts of the scroll are more likely happen at the end of the scroll, not in the middle."

We added a clarification at the beginning of p. 3 of the paper, namely that the Stegemann method itself only tries to estimate the length of the scroll from the preserved scroll inwards. This is an explicit assumption of the “geometric” method that we are analyzing.

1. "Figure 1A is not relevant to the current study: we are not interested in the width of the scroll, but its length. Therefore, the side picture that shows concentric circles must be show."

We moved fig. 1 (now fig. 6) near to its relevant discussion, and added an explanation of the reason for including it, which is to show the different repeating patterns of damages before and after the scroll was unrolled.

2. "Figure 1B and Figure 1C are confusing. If the scroll can be unfolded as seen, there is no problem of measuring its length because the lateral damage does not affect the length. Are the fragments (2*pi rolls) are connected? It looks they are. But if they are, there is no problem of measuring the length of the scroll. If they are not connected the gaps must be shown. How lengths of the gaps were computed? There is some explanation on page 6 on lines 213-222 but they are very short and unclear. Are the gaps were estimated from the wedge approximation in Appendix? If so, it must be clearly stated. I believe that most errors come from how the gap’s length was estimated."

We also added some clarifications for our intentions in examining this scroll. The length of extant scroll is known more or less; that was not the point. Rather, the question is whether examining part of the damage pattern can yield that length.

3. "Abbreviations like 11Q5 (=11QPs) are not spelled out."

We added full names and explanation of abbreviations on p. 4.

We would be most happy to provide any further clarifications, if needed.

Thank you very much.

Sincerely,

Eshbal Ratzon and Nachum Dershowitz
